# Prevalence of non-*Plasmodium falciparum* species in southern districts of Brazzaville in The Republic of the Congo

**DOI:** 10.1186/s13071-022-05312-9

**Published:** 2022-06-16

**Authors:** Jacques Dollon Mbama Ntabi, Abel Lissom, Jean Claude Djontu, Steve Diafouka-Kietela, Christevy Vouvoungui, Reauchelvy Kamal Boumpoutou, Jolivet Mayela, Daniel Nguiffo-Nguete, Francis Nongley Nkemngo, Cyrille Ndo, Romaric Akoton, Romuald Agonhossou, Arsène Lenga, Stravensky Terence Boussougou-Sambe, Luc Djogbénou, Charles Wondji, Ayola Akim Adegnika, Steffen Borrmann, Francine Ntoumi

**Affiliations:** 1 Fondation Congolaise Pour la Recherche Médicale, Brazzaville, Republic of Congo; 2 Faculté des Sciences et Techniques, Université Marien Ngouabi, Brazzaville, Republic of Congo; 3 Institute of Tropical Medicine, University of Tübingen, Tübingen, Germany; 4 Department of Biological Science, Faculty of Science, University of Bamenda, Bamenda, Cameroon; 5 Fondation Pour la Recherche Scientifique (FORS), Institut des Sciences Biomédicales Appliquées (ISBA), BP 88 Cotonou, Benin; 6 Centre de Recherches Médicales de Lambaréné, Lambaréné, Gabon; 7 German Center of Infection Research (DZIF), Tübingen, Germany; 8 Department of Parasitology and Medical Entomology, Centre for Research in Infectious Diseases (CRID), Yaounde, Cameroon; 9 Department of Vector Biology, Liverpool School of Tropical Medicine, Pembroke Place, Liverpool, L3 5QA UK; 10 Tropical Infectious Diseases Research Centre (TIDRC), University of Abomey-Calavi, Cotonou, Benin; 11 Department of Microbiology and Parasitology, University of Buea, Buea, Cameroon; 12 Department of Biological Sciences, Faculty of Medicine and Pharmaceutical Sciences, University of Douala, Douala, Cameroon

**Keywords:** Malaria, Republic of Congo, Non-*Plasmodium falciparum* species

## Abstract

**Background:**

Although *Plasmodium falciparum* infection is largely documented and this parasite is the main target for malaria eradication, other *Plasmodium* species persist, and these require more attention in Africa. Information on the epidemiological situation of non-*P. falciparum* species infections is scarce in many countries, including in the Democratic Republic of the Congo (hereafter Republic of the Congo) where malaria is highly endemic. The aim of this study was to determine the prevalence and distribution of non-*P. falciparum* species infections in the region south of Brazzaville.

**Methods:**

A cross-sectional survey was conducted in volunteers living in rural and urban settings during the dry and rainy seasons in 2021. Socio-demographic and clinical parameters were recorded. *Plasmodium* infection in blood samples was detected by microscopic analysis and nested PCR (sub-microscopic analysis).

**Results:**

Of the 773 participants enrolled in the study, 93.7% were from the rural area, of whom 97% were afebrile. The prevalence of microscopic and sub-microscopic *Plasmodium* spp. infection was 31.2% and 63.7%, respectively. Microscopic *Plasmodium malariae* infection was found in 1.3% of participants, while sub-microscopic studies detected a prevalence of 14.9% for *P. malariae* and 5.3% for *Plasmodium ovale*. The rate of co-infection of *P. malariae* or *P. ovale* with *P. falciparum* was 8.3% and 2.6%, respectively. Higher rates of sub-microscopic infection were reported for the urban area without seasonal fluctuation. In contrast, non-*P. falciparum* species infection was more pronounced in the rural area, with the associated risk of the prevalence of sub-microscopic *P. malariae* infection increasing during the dry season.

**Conclusion:**

There is a need to include non-*P. falciparum* species in malaria control programs, surveillance measures and eradication strategies in the Republic of the Congo.

**Graphical Abstract:**

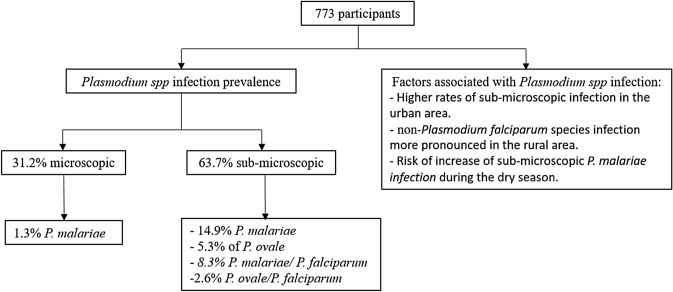

**Supplementary Information:**

The online version contains supplementary material available at 10.1186/s13071-022-05312-9.

## Background

Malaria is a life-threatening disease caused by parasites that are transmitted to people through the bites of infected female *Anopheles* mosquitoes. The WHO estimated that in 2020 there were 241 million malaria cases in 85 malaria endemic countries worldwide, an increase from 227 million in 2019, mainly in countries in the WHO African Region [1]. Also, malaria deaths increased by 12% compared with 2019, to an estimated 627,000; an estimated 47,000 (68%) of the additional 69,000 deaths were due to service disruptions during the COVID-19 pandemic [1].

Among the *Plasmodium* species infecting humans in Africa, *Plasmodium falciparum* is the major cause of malaria cases (99.7%) [2], with a minor but underestimated prevalence of other *Plasmodium* species [3]. *Plasmodium vivax* is the predominant parasite in the WHO region of Americas, causing 75% of malaria cases [4]. Although few studies have identified *P. vivax* infections in Mali and Nigeria [5–7], this *Plasmodium* species remains non-endemic in West and Central Africa because of the lack of the Duffy antigen receptor for chemokines, which is essential for erythrocyte invasion by the parasite [8]. *Plasmodium ovale* infection represents < 1% of all malaria infections worldwide [9, 10]. Despite its low prevalence, however, *P. ovale* infections are directly linked to the danger of relapses after months or even years due to the presence of hypnozoites [11, 12], which may cause severe disease and even death [13]. The global distribution of *Plasmodium malariae* is sparse and variable, but frequently co-endemic with *P. falciparum* [14, 15]. A low prevalence of *P. malariae* infection is commonly reported with asymptomatic cases, however patients may occasionally develop severe symptoms [16]. In addition, the recrudescence of *P. malariae* blood-stage parasites can occur for long periods, even after the infected person has left endemic regions [17, 18].

Despite the decrease in malaria-related morbidity since 2016, the burden of malaria in the Democratic Republic of the Congo (hereafter Republic of the Congo) remains high, with *P. falciparum* as the most prevalent malaria-causing species. A systematic review of the epidemiology revealed that 37% of Congolese living in urban areas tested positive for microscopic *P. falciparum* infection compared to 59% in the peri-urban areas, with these populations showing 11% and 20% submicroscopic infections, respectively [19]. However, recent information on the epidemiology of non-*Plasmodium falciparum* species, mainly *P. malariae* and *P. ovale*, is limited. Taking into account the new malaria elimination strategies based on the interruption of local transmission of human *Plasmodium* [20], intensification of malaria species surveillance has been strongly recommended. Therefore, the aim of the present study was to determine the prevalence and the distribution of *P. malariae* and *P. ovale* infection, based on multiple community surveys in rural and urban settings in Brazzaville, the capital of the Republic of the Congo and home to > than 50% of the Congolese population.

## Methods

### Study areas

**Fig. 1:**
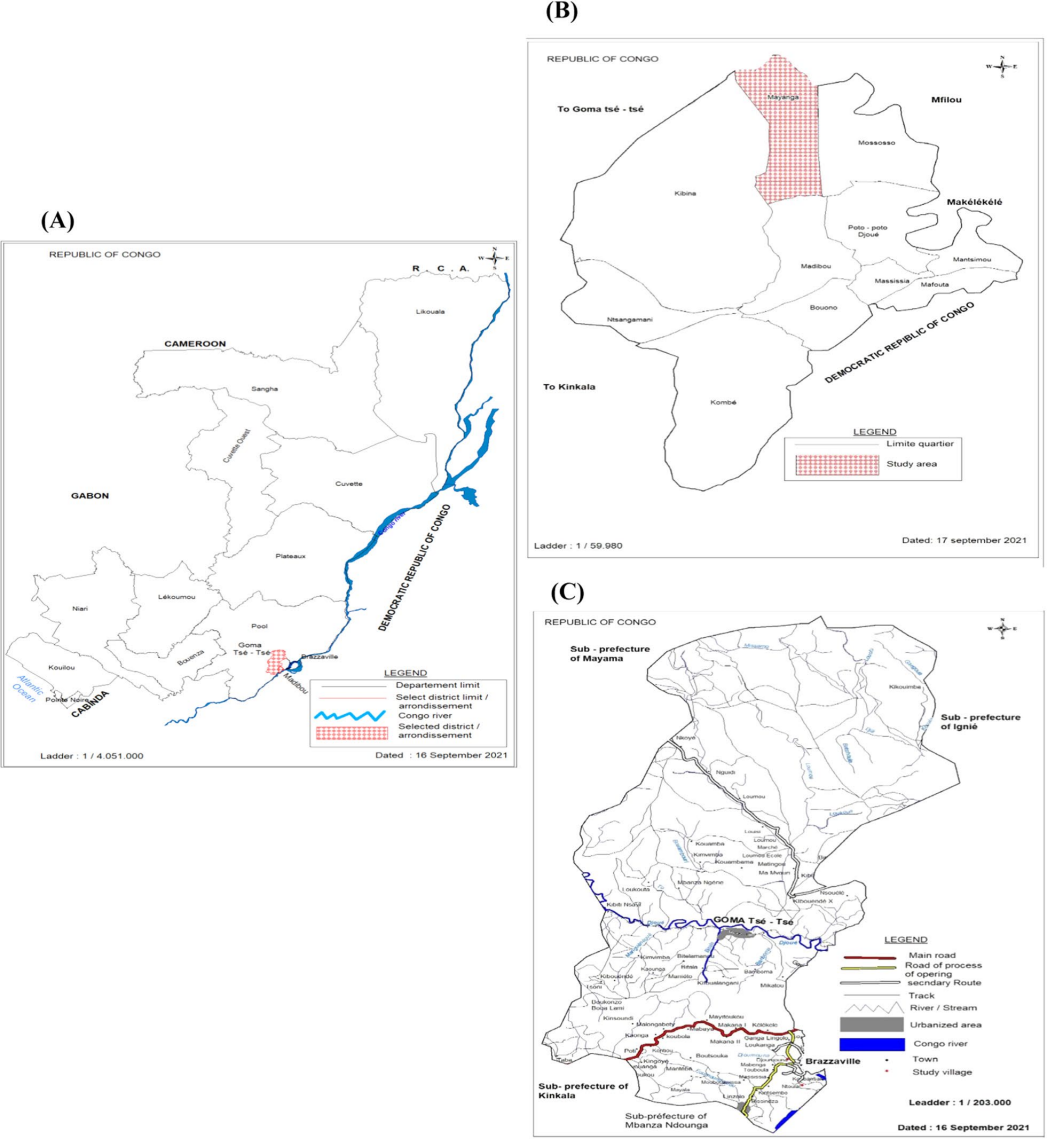
Map of the Republic of the Congo (**a**) showing the localities surveyed in Goma Tsé-Tsé District (**b**) and Madibou District (**c**). In Goma Tsé-Tsé District, the red dots represent the selected villages (Ntoula and Djoumouna), and in Madibou district the red striped area represents the urban area (Mayanga) selected for the survey

The study was conducted in rural areas of the Goma Tsé-Tsé District (Ntoula and Djoumouna villages) in the Department of Pool and in the urban area of Brazzaville Department (Mayanga in the West Quarter of Madibou District) of the Republic of the Congo (Fig. 1). The two departments have a tropical humid climate divided in two seasons: a short dry season (June to September) and a long rainy season (October to May). The geo-location of these zones is 4.36° S, 15.15° E, and the average altitude is 217 m a.s.l [21]. Djoumouna is a village located 25 km from Brazzaville. It is characterized by the presence of a gallery forest bordering the Djoumouna River. The locality is surrounded by four rivers (Lomba, Kinkoue, Loumbangala and Djoumouna rivers) which supply water to a series of fish farming ponds [22] that can serve as potential foci of malaria vectors. The population of Djoumouna village comprises about 800 inhabitants, with agriculture as the main economic activity. Ntoula is a neighboring village of Djoumouna and irrigated by two rivers. This village has around 635 inhabitants who mainly depend on agriculture and fishing for their livelihoods. Mayanga is an urban area located in the 8th Madibou District in the south of Brazzaville. With a population of about 28,422 inhabitants, Mayanga is characterized by the presence of market gardening sites (Agni-Congo 1 and 2 and the Groupement Jean Felicien Mahouna). Irrigation water is supplied by three rivers (Djoué, Laba and Matou rivers). Several public and private services are established in Mayanga, such as health centers and primary and secondary schools.

### Study design

A cross-sectional survey was carried out from March to September 2021, covering both the rainy and dry seasons. Individuals aged at least 1 year who lived in the study areas for at least 3 months were included in the study. Volunteers from a random sample of households were recruited in each area. Participants who decided to withdraw their consent during the study were excluded from the analysis. Socio-demographic and clinical data of the participants were recorded in a well-structured collection sheet (age, gender, temperature, bed net use etc.). The axillary temperature was determined to define fever in the participants. Fever was diagnosed when the axillary temperature was ≥ 37.5 °C [23, 24]. Bed net use was defined based on the answers of participants, and on the observation of bed nets in each household. After an examination by a clinician (diagnosis of fever, headaches, vomiting, nausea etc.), blood samples (3 ml) were collected in EDTA tubes by a nurse and the tubes then transported to the “Centre de Recherches sur les Maladies Infectieuse-Christophe Merieux” (CeRMI) for further investigations, including thick and thin blood smears for microscopic examination. Participants presenting a positive blood smear were treated by the medical staff with artemisinin-based combination therapy (ACT) in accordance to WHO and national malaria program policies [25]. Hemoglobin level was determined performed using CYANHemato, a fully automated bench-top hematology cell counter (Cypress Diagostic, Hulshout, Belgium).

### Microscopic screening of *Plasmodium* infections

*Plasmodium* infection was screened by microscopy using the Lambaréné method [26]. In brief, 10 µl of whole blood of each sample was distributed on a microscope slide over a rectangular area of 10 × 8 mm. The contour of the rectangle was drawn on a piece of paper placed under the slide before the whole blood sample was spread. The slides were air dried and stained for 20 min with 20% Giemsa solution (Giemsa R-Solution [Merck, Darmstadt, Germany], Titrisol buffer pH 7.2 [Merck]). Each slide was read by two microscopists. When no agreement was reached on a specific slide, a third microscopist was asked to decide. Positive slides of each parasite species served as the quality controls of the microscopy studies. The number of parasites and the number of high-power fields (HPF) were counted, and parasitemia per microliter was calculated using the microscope factor of 708 as follows:

$${\text{Parasiteamia }}\left( {{{{\text{para}}} \mathord{\left/ {\vphantom {{{\text{para}}} {\mu {\text{l}}}}} \right. \kern-\nulldelimiterspace} {\mu {\text{l}}}}} \right)\,\frac{{{\text{N}}_{{{\text{Para}}}} }}{{{\text{N}}_{{{\text{HPF}}}} }} \times {\text{Microscope Factor}},$$

where N is the number and para represents parasites.

### *Plasmodium* species identification by PCR

The parasite’s DNA was extracted from 200 µl whole blood samples using the QIAamp DNA mini Kit (Qiagen, Hilden, Germany) according to the manufacturer’s instructions. DNA was recovered in 150 µl of elution buffer and stored at − 20 °C until use.

The detection of malaria parasite DNA was based on nested PCR amplification of the* 18S* ribosomal RNA (rRNA) gene in a total reaction volume of 20 µl using the appropriate primers (Additional File 1: Table S1). The first PCR reaction targeted the *Plasmodium *spp. and was performed in a reaction volume composed of 18 µl of a master mix (2.5 µl of 10× PCR buffer, 1.25 µl of 25 mM MgCl_2_, 0.5 µl of 10 mM dNTPs, 0.5 µl of each 10 µM primer, 0.25 µl of *Taq* DNA polymerase 5U/µl and 12.5 µl of nuclease-free water) and 2 μl of the extracted DNA. The reaction was carried out under the following cycling conditions: an initial denaturation at 94 °C for 4 min; followed by 35 cycles of denaturation at 94 °C for 30 s, annealing at 55 °C for 1 min and extension at 72 °C for 1 min, with a final extension at 72 °C for 4 min. In the second PCR, 19 µl of master mix (containing 13.5 µl of nuclease-free water and the same volume of reagents as described for the first PCR) and 1 µl of primary PCR product were added to the PCR tube, and the reaction was carried out under the same cycling conditions as described for the first PCR, except that the annealing temperature was 58 °C.

PCR products and the 100-bp molecular weight maker were stained with Syber Green solution (1:1, v/v), electrophesed at 100 V for 45 min in a 1.5% agarose gel and visualized on the GelDoc™ EZ Imager (Bio-Rad Laboratories, Hercules, CA, USA). A sample was considered positive for *P. falciparum*, *P. malaria*,* P. ovalea* or *P. Vivax* if a 205-bp, 144-bp, 800-bp or 120-bp band was detected, respectively. The known *Plasmodium*-positive samples from our library and distilled water served as positive and negative controls, respectively, in every set of reactions.

### Statistical analysis

Management and tabulation of raw data were carried out using Microsoft Excel (Microsoft Inc., Redmond, WA, USA) version 2016. All statistical analyses were performed using SPSS version 22.1 (SPSS, IBM Corp., Armonk, NY, USA). The normality of data distribution was checked using the Shapiro–Wilk test [27]. Variables were expressed as proportions for categorical variables or as medians (with range)/means (standard deviations [SD}) for continuous variables. The prevalence of *Plasmodium* spp. infections was determined as the proportion of individuals identified as positive for the presence of parasites (either for all parasite species or for an individual species) in each community. Age was stratified into five groups according to WHO guidelines [28]: 1–4, 5–9, 10–14, 15–19 and ≥ 20 years.

Participants with a negative result in the microscopic examination, but positive in the PCR analysis were classified as the sub-microscopically infected group. Those who had microscopic* Plasmodium*-positive results were classified as the microscopically infected group.

Chi-square tests (or Fisher’s exact tests when appropriate) were used to calculate the differences in participant characteristics and prevalences among groups, and to assess associations between independent variables and* Plasmodium* spp infection. The strength of association between each risk factor and the occurrence of Plasmodium infection was calculated using univariate logistic regression. The relative risks (RR) and 95% confidence intervals (CIs) were calculated to determine the risk factors associated with *Plasmodium* infection. The level of significance was set at *P* < 0.05.

### Ethical consideration

This study received ethical approval from the Institutional Ethics Committee of “Fondation Congolaise pour la Recherche Medical” (No. 013/CIE/FCRM/2018), administrative authorizations from the Marien Ngouabi University (No. 247/UMNG.FST.DFD.FD-SBIO) and approval from the major of each study area. Prior to enrollment, participants were informed in writing and orally about the study and its benefits. Before enrollment, a consent form was signed by each participant or by the parent of a participant who was a minor (< 18 years). Children aged between 15 to 17 years signed an assent form.

## Results

### Characteristics of the study population

Of the 773 participants enrolled in this study 573 and 200 were resided in Goma Tsé-Tsé District (District 1) and Madibou District (District 2), respectively (Table 1). The age of the participants ranged from 1 to 88 years, with a median age of 15 (range: 1–88) years in Goma Tsé-Tsé District and 16 (range: 2–81) years in Madibou District. There were more female than male participants in both districts (Goma Tsé-Tsé: 54.5% [312/573]; Madibou: 59.5% [119/200]). Of the five age groups, participants aged > 20 years formed the largest group in both districts (Goma Tsé-Tsé: 41.5% [238/573]; Madibou: 45.5% [91/200]). A total of 42 of the 773 participants (5.4%) presented an increased axillary temperature**.** Almost all study participants slept under bed nets in both study areas.

**Table 1:** Demographic characteristics of the study population

Characteristics	Districts (*n* = 773)		Total (*n* = 773)
	District 1 (*n* = 573)	District 2 (*n* = 200)	
Season,%(n)			
Rainy	51.0 (292)	44.0 (88)	49.2 (380)
Dry	49.0 (281)	56.0 (112)	50.8 (393)
Age, years, median (Max–Min)	15 (1–88)	16 (2–81)	15 (1–88)
Age groups, years,% (n)			
1–4	10.1 (58)	8.0 (16)	9.6 (74)
5–9	22 (126)	18.5 (37)	21.1 (163)
10–14	17.8 (102)	18.5 (37)	18.0 (139)
15–19	8.6 (49)	9.5 (19)	8.8 (68)
≥ 20	41.5 (238)	45.5 (91)	42.5 (329)
Gender, % (*n*)			
Female	54.5 (312)	59.5 (119)	55.8 (431)
Male	45,5 (261)	40,5 (81)	44.2 (342)
Fever, % (*n*)			
Yes (≥ 37.5 °C)	6.3 (36)	3 (6)	5.4 (42)
No (< 37.5 °C)	93.7 (537)	97 (194)	94.6 (731)
Geometric mean (95% CI) of parasitemia (parasite/µl)^a^			
Afebrile cases	276.8 (205.4—373.1)	161.5 (84.4—309.2)	
Febrile cases	443.1 (134.5—1460)	78.2 (5027—1.215e + 006)	
Hemoglobin, g/dl, % (*n*)			
< 7	1.2 (7)	1.0 (2)	1.2 (9)
7–10	10.7 (61)	5.5 (11)	9.3 (72)
> 10	88.1 (505)	93.5 (187)	89.5 (692)
Bed net use, % (*n*)			
Yes	97.4 (558)	97.5 (195)	97.4 (753)
No	2.6 (15)	2.5 (5)	2.6 (20)

The table shows the general characteristics of the participants in the two study areas: Goma Tsé-Tsé District (District 1; 573 participants) and Madibou District (District 2; 200 participants)

*CI* Confidence interval, *Max–Min* maximum-minimum, *n* number of participants,

^a^Febrile: participants with a temperature ≥ 37.5 °C. Afebrile: participants with a temperature < 37.5 °C

### Microscopic *Plasmodium* spp. infection

**Fig. 2:**
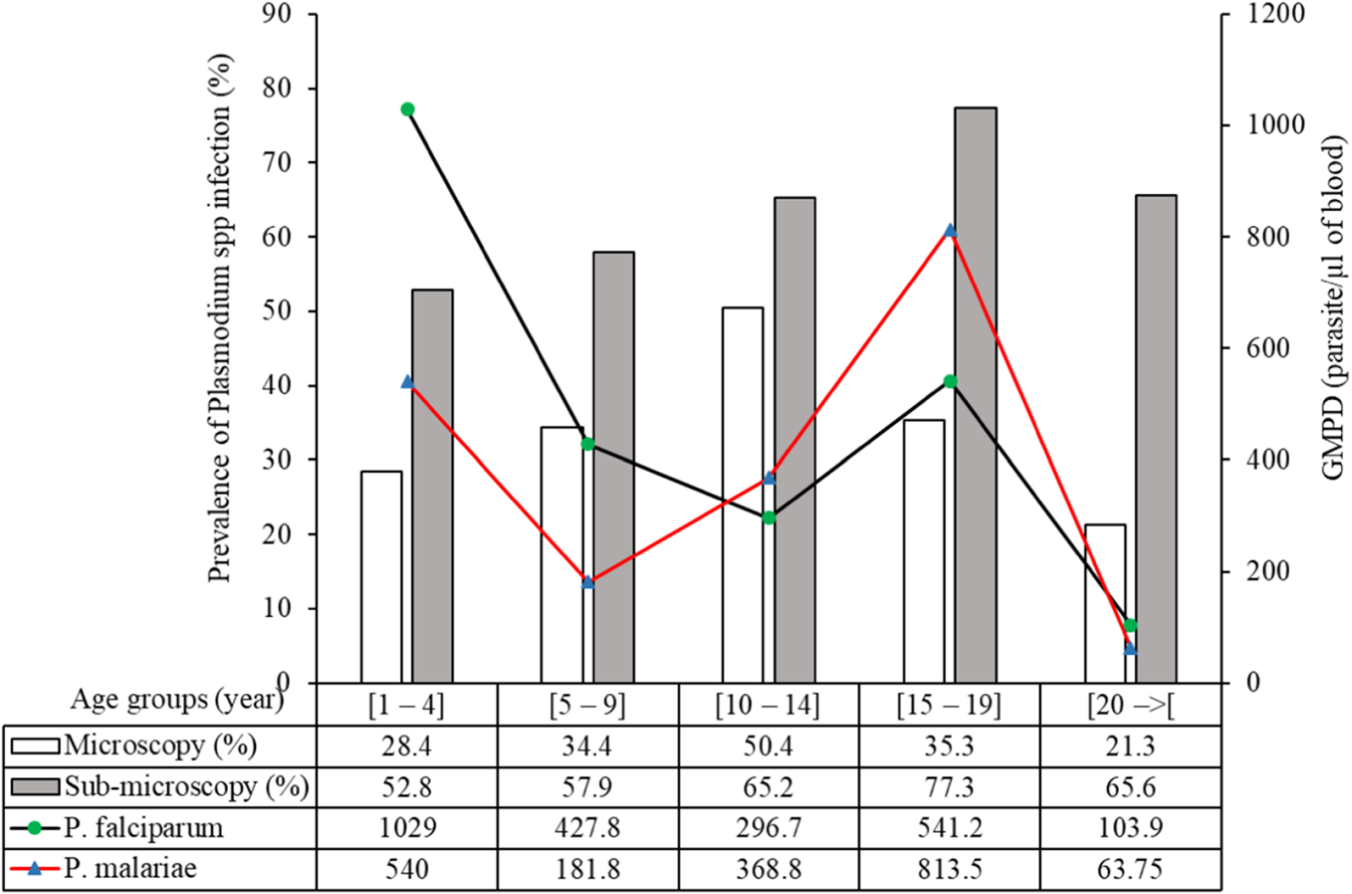
Age-specific prevalence of *Plasmodium* spp. infection in participants based on microscopy and sub-microscopy studies. Abbreviations: GMPD, Geometric mean of parasite density

The overall prevalence of *Plasmodium* spp. was 31.2% (241/773) (Table 2). Among the identified *Plasmodium* species, *P. falciparum* was detected in 30.7% (237/773) of the study population (including 29.9% [231/773] as mono-infection), while *P. malariae* was prevalent at a level of 1.3% (10/773) individuals (including 0.5% [4/773] as mono-infection). The rate of *P. falciparum*/*P. malariae* co-infection was 0.8% (6/773). According to the districts, the rate of positive microscopic infection was significantly (*P* < 0.0001) higher in Goma Tsé-Tsé (35.1%; 201/573) than in Madibou (20.0%; 40/200). *Plasmodium malariae* was identified in 1.4% (8/573) of the participants living in Goma Tsé-Tsé District with 0.7% (4/573) of cases co-infected or not with *P. falciparum*. *Plasmodium malariae* was detected in 1% (2/200) of the participants living in Madibou as co-infection with *P. falciparum*. Although the prevalence of microscopic *Plasmodium * spp. infection was low in participants aged < 5 years (28.4%; 21/74), parasite density in children in this age group was higher both for *P. falciparum* and *P. malariae* than in children aged between 6 and 14 years (Fig. 2).

**Table 2:** Prevalence of *Plasmodium* species identified by microscopy and by sub-microscopy

*Plasmodium* spp. infections	Goma Tsé-Tsé District	Madibou District	Overall
Plasmodium species by microscopy study (*n*_1_ = 573; *n*_2_ = 200; (*N* = 773)			
Overall positivity, % (*n*)	35.1 (201)	20.0 (40)**	31.2 (241)
* P. falciparum*, % (*n*)	33.7 (193)	19 (38)	29.9 (231)
* P. malariae*, % (*n*)	0.7 (4)	0 (0)	0.5 (4)
* P. falciparum*/*P. malariae*, % (*n*)	0.7 (4)	1 (2)	0.8 (6)
Plasmodium species by sub-microscopy study (*n*_1_ = 373; *n*_2_ = 159; (*N* = 532)			
Overall positivity, % (n)	59.8 (228)	73.0 (116)**	63.7 (339)
Mono-infection			
* P. falciparum*	41.0 (153)	56.0 (89)	45.5 (242)
* P. malariae*	5.1 (19)	3.8 (6)	4.7 (25)
* P. ovale*	1.1 (4)	0.0 (0)	0.8 (4)
Co-infection			
* P. falciparum/P. malariae*	7.8 (29)	9.4 (15)	8.3 (44)
* P. falciparum/P. ovale*	2.7 (10)	2.5 (4)	2.6 (14)
* P. malariae/P. ovale*	0.3 (1)	0 (0)	0.2 (1)
* P. falciparum/P. malariae/P. ovale*	1.9 (7)	1.3 (2)	1.7 (9)

Prevalence of *Plasmodium* species was compared between the Goma Tsé-Tsé and Madibou districts

The Chi^2^ test was used to compare the proportion of cases between the two districts.

*N* Overall number of participants, *n*_1_, *n*_2_ total number of participants in Goma Tsé-Tsé and Madibou districts, respectively

**Significant difference in prevalence between the two districts according to overall microscopic findings at *P* < 0.0001 and according to sub-microscopic study at *P* = 0.0099

### Sub-microscopic *Plasmodium* spp. infection

Sub-microscopic *Plasmodium* spp. infections were found in 63.7% (339/532) of all samples (Table 2). Regardless of co-infections*, P. falciparum*, *P. malariae* and *P. ovale* were detected by PCR in 58.1% (309/532), 14.9% (79/532) and 5.3% (28/532) of participants, respectively. Dual* Plasmodium* infection with *P. falciparum/P. malariae* accounted for 8.3% (44/532) of *Plasmodium* spp. infections, while dual infections with *P. falciparum/P. ovale* and *P. malariae/P. ovale* accounted for 2.6% (14/532) and 0.2% (1/532), respectively. Triple infections were detected in 1.7% of all participants.

According to study site, sub-microscopic *Plasmodium* spp. infection was significantly higher (*P* = 0.0099) in Madibou (72.5%; 116/160) than in Goma Tsé-Tsé (59.9%; 223/372). A similar trend was observed for the prevalence of mono-infection by *P. falciparum* (Madibou: 56% (89/160); Goma Tsé-Tsé: 41% [153/372]), while the reverse was observed for *P. malariae* infection (Goma Tsé-Tsé: 5.1% [19/372]; Madibou: 3.8% [6/160]) and *P. ovale* infection (Goma Tsé-Tsé: 1.1% [4/372]; Madibou: 0.0% [0/160]). The prevalence of sub-microscopic infection was high in all age groups (Fig. 2), with study participants between the ages of 15 and 19 years harboring a highest proportion of sub-microscopic infection (77.3%).

### Predictors of microscopic malaria infection

The relationship between participant characteristics and the presence of *Plasmodium* spp. infections was investigated. Those participants found to be anemic were classified according to their age group following the WHO guideline [29]. *Plasmodium falciparum* infection was significantly associated with anemia in children aged < 15 years (*P* = 0.0374 for children aged 1–4 years, *P *= 0.0001 for 5- to 9-year olds,* P* < 0.0001 for 10–to 14-year olds), but no relationship was observed between *P. malariae* infection and the risk of anemia (Table 3). As shown in Table 4, the rainy season was shown to be a risk factor (RR: 3.1, 95% CI: 1.7–5.7; *P* = 0.0003) for increased *P. falciparum* prevalence in Madibou. *Plasmodium falciparum* infection was associated with gender in Goma Tsé-Tsé (RR: 1.4, 95% CI: 1.1–1.7; *P* = 0.0072), but no such association was found in Madibou. According to the age group, *P. falciparum* prevalence for all age group was higher in Goma Tsé-Tsé District than in Mabidou District, with participants aged 5 to 14 years having a higher risk of malaria infection. The presence of fever was significantly (*P* = 0.0223 [Goma Tsé-Tsé]; *P* = 0.0306 [Madibou]) associated with *P. falciparum* infection in the two districts. No association was observed between the socio-demographic characteristics of the study population and the microscopic prevalence of *P. malariae* in the two districts (Table 5).

**Table 3:** Association of anemia with microscopic* Plasmodium* spp. infection according to age groups

Age groups (years)	Normal values (g/dl)	Anemic, % (*n*)	RR (95% CI)	*P*-value
*P. falciparum infection*				
1–4 (*N* = 43)	≥ 11	39.5 (17)	0.637 (0.417–0.974)	0.0374
5–9 (*N* = 81)	≥ 11.5	46.9 (38)	0.537 (0.393–0.733)	0.0001
10–14 (*N* = 86)	≥ 12	58.1 (50)	0.433 (0329–0.571)	< 0.0001
≥ 15 (*N* = 262)	≥ 13	25.2 (66)	Ref. group	–
*P. malariae infection*				
1–4 (*N* = 43)	≥ 11	2.3 (1)	0.492 (0.052–4.626)	0.5353
5–9 (*N* = 81)	≥ 11.5	2.5 (2)	0.464 (0.079–2.727)	0.3953
10–14 (*N* = 86)	≥ 12	3.5 (3)	0.328 (0.068–1.596)	0.1674
≥ 15 (*N* = 262)	≥ 13	1.1 (3)	Ref. group	–

*n* Number of infected participants who were anemic, *N* total number of anaemic participants,* RR* relative risk

**Table 4:** Association of socio-demographic characteristics with microscopic *P. falciparum* infection

Characteristics	Total number	Goma Tsé-Tsé		*P*-value	Total number	Madibou		*P*-value
		*P. falciparum* infection				*P. falciparum* infection		
		% (*n*)	RR (95% CI)			% (*n*)	RR (95% CI)	
Overall	573	34.4 (197)	–	–	200	20.5 (41)	–	–
*Seasons*								
Rainy	292	37.0 (108)	1.2 (0.9–1.5)	0.1821	88	33.0 (29)	3.1 (1.7–5.7)	0.0003
Dry	281	31.7 (89)			112	12.0 (12)		
*Gender*								
Female	312	29.5 (92)	1.4 (1.1–1.7)	0.0072	119	18.5 (22)	1.3 (0.7–2.2)	0.392
Male	261	40.2 (105)			81	23.5 (19)		
*Age group (years)*								
1–4	58	34.5 (20)	1.5 (1.0–2.2)	0.076	16	6.3 (1)	Ref. group	-
5–9	126	34.9 (44)	1.5 (1.1–2.1)	0.0192	37	29.7 (11)	4.8 (0.7–33.8)	0.1191
10–14	102	58.8 (60)	2.5 (1.9–3.3)	< 0.0001	37	24.3 (9)	3.9 (0.5–28.2)	0.1788
15–19	49	34.4 (17)	1.5 (0.9–2.3)	0.0888	19	31.6 (6)	5.1 (0.7–37.7)	0.1142
≥ 20	238	23.3 (56)	Ref. group	-	91	15.4 (14)	2.5 (0.3–17.4)	0.3672
*Fever*								
Yes (≥ 37.5 °C)	36	50.0 (18)	1.5 (1.1–2.1)	0.0223	6	50.0 (3)	2.6 (1.1–6.0)	0.0306
No (< 37.5 °C)	537	33.3 (179)			194	19.6 (38)		
*Bed net use*								
Yes	558	34.2 (191)	1.2 (0.6–2.2)	0.6281	195	25.8 (40)	1.0 (0.2–6.1)	0.9777
No	15	40.0 (6)			5	20.0 (1)		

*RR (95%CI) * relative risk (95% Confidence interval), *n* Number of participants, % percentage, *Nber* number

**Table 5:** Association of socio-demographic characteristics with microscopic *P. malariae* infection

Characteristics	Total number	Goma Tsé-Tsé		*P*-value	Total number	Madibou		*P*-value
		*P*. *malariae* infection				*P*. *malariae* infection		
		% (*n*)	RR (95% CI)			% (*n*)	RR (95% CI)	
Overall	573	1.4(8)	–		200	1.0 (2)	–	
*Seasons*								
Rainy	292	2.1 (6)	2.9 (0.5–14.2)	0.1918	88	0.0 (0)	0.3 (0.0 –5.2)	0.374
Dry	281	0.7 (2)			112	1.8 (2)		
*Gender*								
Female	312	1.6 (5)	1.4 (0.3–5.8)	0.6469	119	0.0 (0)	0.1 (0.0 –2.8)	0.197
Male	261	1.1 (3)			81	2.5 (2)		
*Age group (years)*								
1–4	58	0.0 (0)	Ref. group	–	16	6.3 (1)	3.5 (0.2–81.1)	0.430
5–9	126	1.6 (2)	2.3 (0.1–47.6)	0.585	37	0.0 (0)	0.5 (0.0–25.5)	0.746
10–14	102	2.9 (3)	4.0 (0.2–76.3)	0.355	37	0.0 (0)	0.5 (0.0–25.5)	0.746
15–19	49	4.1 (2)	5.9 (0.3–120.0)	0.248	19	0.0 (0)	Ref. group	-
≥ 20	238	0.4 (1)	0.7 (0.0–17.9)	0.853	91	1.1 (1)	0.7 (0.0–15.4)	0.791
*Fever*								
Yes (≥ 37.5 °C)	36	2.8 (1)	2.1 (0.3–16.8)	0.473	6	0.0 (0)	5.6 (0.3–105.5)	0.252
No (< 37.5 °C)	537	1.3 (7)			194	1.0 (2)		
*Bed net use*								
Yes	558	1.4 (8)	0.5 (0.0 –8.1)	0.615	195	1.0 (2)	0.2 (0.0 –2.9)	0.209
No	15	0.0 (0)			5	0.0 (0)		

*n* Number of participants

## Predictors of sub-microscopic malaria infection

In Madibou District, the risk of sub-microscopic *P. falciparum* infection increased during the rainy season (RR: 1.3, 95% CI: 1.0—1.5; *P* = 0.020), and in the absence of bed net use. Participants aged between 15 and 19 years were at the highest risk (RR: 1.5, 95% CI: 1.0—2.1;  *P* =  0.041) of sub-microscopic *P. falciparum* infection in Goma Tsé-Tsé District (Table 6). In the Goma Tsé-Tsé area, individuals were at a higher risk (RR: 1.7, 95% CI: 1.0—2.8; *P* = 0.045) of sub-microscopic *P. malariae* infection during the dry season. No risk factor was identified for the presence of sub-microscopic *P. ovale* infection (Tables 7, 8).

**Table 6:** Association of socio-demographic characteristics with sub-microscopic *P. falciparum* infection detected by PCR

Characteristics	Total number	Goma Tsé-Tsé		*P*-value	Total number	Madibou		*P*-value
		*P. falciparum* infection				*P. falciparum* infection		
		% (*n*)	RR (95% CI)			% (*n*)	RR (95% CI)	
Overall	373	53.4 (199)	–		159	69.2 (110)	–	
*Seasons*								
Rainy	180	48.3 (87)	0.8 (0.–1.0)	0.063	59	79.7 (47)	1.3 (1.–1.5)	0.020
Dry	193	58.0 (112)			100	63.0 (63)		
*Gender*								
Female	220	50.9 (112)	1.1 (0.–1.3)	0.252	97	71.1 (69)	0.9 (0.–1.2)	0.513
Male	153	56.9 (87)			62	66.1 (41)		
*Age group (years)*								
1–4	38	47.4 (18)	1.1 (0.7–1.6)	0.763	15	46.7 (7)	Reference group	–
5–9	81	44.4 (36)	Reference group	–	26	76.9 (20)	1.6 (0.–2.9)	0.092
10–14	41	56.1 (23)	1.3 (0.–1.8)	0.210	28	67.9 (19)	1.5 (0.–2.6)	0.220
15–19	31	64.5 (20)	1.5 (1.–2.1)	0.041	13	76.9 (10)	1.6 (0.–3.1)	0.113
≥ 20	182	56.0 (102)	1.3 (1.–1.7)	0.099	77	68.8 (53)	1.5 (0.–2.6)	0.175
*Fever*								
Yes (≥ 37.5 °C)	17	52.9 (9)	1.0 (0.–1.6)	0.972	3	(1)	0.5 (0.–2.4)	0.366
No (< 37.5 °C)	356	53.4 (190)			156	(109)		
*Bed net use*								
Yes	364	(194)	1.0 (0.–1.7)	0.891	155	68.4 (106)	1.5 (1.–1.6)	< 0.0001
No	9	(5)			4	100 (4)		

*n *Number of participants

**Table 7:** Association of socio-demographic characteristics and sub-microscopic *P*. *malariae* infection detected by PCR

Characteristics	Total number	Goma Tsé-Tsé		*P*-value	Total number	Madibou		*P*-value
		*P*. *malariae* Infection				*P*. *malariae* Infection		
		%(*n*)	RR (95% CI)			%(*n*)	RR (95% CI)	
Overall	373	15.0 (56)	–		159	77.3 (123)	–	
*Seasons*								
Rainy	180	11.1 (20)	1.7 (1.0–2.8)	0.045	59	18.6 (11)	1.6 (0.7–3.3)	0.251
Dry	193	18.7 (36)			100	12.0 (12)		
*Gender*								
Female	220	14.5 (32)	1.1 (0.7–1.8)	0.761	97	14.4 (14)	1.0 (0.5–2.2)	0.988
Male	153	15.7 (24)			62	14.5 (9)		
*Age group (years)*								
1–4	38	10.5 (4)	1.0 (0.3 –2.9)	0.924	15	13.3 (2)	1.7 (0.3–11.1)	0.561
5–9	81	11.1 (9)	Ref. group	-	26	7.7 (2)	Ref. group	–
10–14	41	12.2 (5)	1.1 (0.4–3.1)	0.859	28	10.7 (3)	1.4 (0.3–7.7)	0.704
15–19	31	22.6 (7)	2.0 (0.8–5.0)	0.121	13	23.1 (3)	3.0 (0.6–15.8)	0.195
≥ 20	182	17.0 (31)	1.5 (0.8 –3.1)	0.228	77	16.9 (13)	2.2 (0.5–9.1)	0.278
*Fever*								
Yes (≥ 37.5 °C)	17	17.6 (3)	1.2 (0.4–3.4)	0.752	3	0.0 (0)	0.8 (0.1–11.5)	0.893
No (< 37.5 °C)	356	14.9 (53)			156	14.7 (23)		
*Bed net use*								
Yes	364	14.8 (54)	0.7 (0.2–2.3)	0.525	155	14.8 (23)	1.5 (0.1–21.4)	0.762
No	9	22.2 (2)			4	0.0 (0)		

*n* Number of participants

**Table 8:** Association of socio-demographic characteristics and sub-microscopic *P*. *ovale* infection detected by PCR

Characteristics	Total number	Goma Tsé-Tsé		*P*-value	Total number	Madibou		*P*-value
		*P*. *ovale* infection				*P*. *ovale* infection		
		% (*n*)	RR (95% CI)			% (*n*)	RR(95% CI)	
Overall	373	5.9 (22)	–		159	3.8 (6)	–	
*Seasons*								
Rainy	180	5.0 (9)	0.7 (0.3–1.7)	0.479	59	3.4 (2)	0.8 (0.2–4.5)	0.845
Dry	193	6.7 (13)			100	4.0 (4)		
*Gender*								
Female	220	6.4 (14)	0.8 (0.4–1.9)	0.648	97	1.0 (1)	7.8 (0.9–65.4)	0.058
Male	153	5.2 (8)			62	8.1 (5)		
*Age group (years)*								
1–4	38	5.3 (2)	1.1 (0.2–7.3)	0.078	15	6.7(1)	2.6 (0.–59.4)	0.544
5–9	81	4.9 (4)	1.0 (0.2–5.3)	0.988	26	3.8 (1)	1.6 (0.–35.8)	0.782
10–14	41	4.9 (2)	Ref. group	–	28	0.0 (0)	0.5 (0.–23.1)	0.712
15–19	31	6.5 (2)	1.3 (0.2–8.9)	0.774	13	0.0 (0)	Ref. group	-
≥ 20	182	6.6 (12)	1.4 (0.3–5.8)	0.685	77	5.2 (4)	0.–28.4)	0.743
*Fever*								
Yes (≥ 37.5 °C)	17	0.0 (0)	0.4 (0.0–7.0)	0.560	3	0.0 (0)	3.0 (0.2–44.9)	0.422
No (< 37.5 °C)	356	6.2 (22)			156	3.8 (6)		
*Bed net use*								
Yes	364	6.0 (22)	1.2 (0.1–18.9)	0.881	155	(6)	0.4 (0.0 –6.4)	0.530
No	9	0.0 (0)			4	0.0 (0)		

*n* Number of participants

## Discussion

Since publication of the systematic review on the malaria situation in the Republic of the Congo in 2016 [19], studies conducted in the country have mostly focused on the epidemiology and diversity of *P. falciparum* infection [30–32]. However, information on the epidemiological situation of non-*Plasmodium falciparum* species in the country is lacking. The aim of the present study was to evaluate the prevalence of *Plasmodium* species in two endemic areas for malaria of the Republic of the Congo, namely Goma Tsé-Tsé District, as representative of rural areas in the Republic of the Congo, and Madibou District, which is an urban area. Of 773 participants enrolled in this study, 573 and 200 were living in Goma Tsé-Tsé District and Madibou District, respectively. This vast difference in number of participants reflects the low interest of the population of Madibou to participate in the study, possibly due to the presence of health centers close by. Almost all study participants were sleeping under bed nets (97.4%), suggesting a strict compliance of the inhabitants of these localities to the malaria control measures.

The overall prevalence of *Plasmodium* spp. detected by microscopy was 31.2% in this study. *Plasmodium falciparum* remained the most prevalent species, in accordance with prevalences reported in many previous studies conducted in African malaria endemic areas [33–36]. The high prevalence of *P. falciparum* in the overall study population demonstrates the importance of malaria control strategies specifically against this species. The prevalence of malaria infection was significantly higher in villages of Goma Tsé-Tsé District than in Madibou District, which may be related to the different ecological situations in these two areas (rural vs. urban) [37]. The prevalence of *Plasmodium* spp. in this study was higher than that reported in recent studies in Benin (23.7%) by our research group [33], but similar to the findings of Amoah et al. (32%) [34].

Among the identified non*-P. falciparum* species in the whole study population, only *P. malariae* was detected by microscopy at a rate of 1.3% (with 0.8% of dual *P. falciparum*/*P. malariae* infection). This prevalence was higher in the rural area of Goma Tsé-Tsé District (1.4%) compared to the urban area of Madibou District (1%), suggesting that this variation in prevalence was probably due to the socio-demographic characteristics of each study area. Suitable containers or conditions for water to pool and persist, deforestation and agriculture have all been associated with an increased risk of malaria transmission [37]. The *P. malariae* prevalence observed in this study was comparable to that reported in Benin**,** in which an increase of this prevalence was observed over the past 10 years [33]. A potential explanation could be the differences in the use of bed nets by the population. Almost all participants of this study, compared to less than one quarter of the study population in Benin, slept under a bed net. This difference highlights a heath preoccupation related to this species among the non*-Plasmodium falciparum* parasites that must be taken into account in the WHO strategic plan for malaria eradication [20].

The rainy season was associated with a higher prevalence of *P. falciparum* infection in Madibou District. In the villages of Goma Tsé-Tsé District, where the prevalence of *P. falciparum* was high and almost stable throughout the year, men and children appeared to be at higher risk of getting malaria infection. All of these results suggest that malaria management strategies must be directly applicable to the area they are being used: in urban areas, they should take seasonality into account; in rural areas, they must be permanent, with a particular focus on the sensitization of men and adolescents. Fever was significantly associated with *P. falciparum* infection in the two districts of this study. We did not observe any association between socio-demographic factors or clinical outcomes with an increased prevalence of *P. malariae*, probably due to low number of events and, therefore, power issues. However, many studies have reported a significant association of *P. malariae* infection with severe anemia, probably due to prolonged erythrocyte destruction and bone marrow suppression with a minimal reduction in erythrocyte life-span at a low parasitemia level [38, 39]. Pulmonary complications and renal impairment have also been reported to be associated with *P. malariae* infection [40]. The pooled proportion of severe complication caused by *P. malariae* infection was reported to be 2% [16].

Compared to microscopic malaria infection, a higher sub-microscopic infection (63.7%) of *Plasmodium* spp. was observed, being more pronounced in older participants (> 15 years). These findings are similar to those reported in a previous study from Ghana (66%) [34], and higher than those reported from a study conducted in Benin [33]. This difference could be attributed to geographical zone, including the level of local *Plasmodium* infection endemicity. Indeed, in Benin, the survey was conducted in villages, while in Ghana, the study covered a mixture of communities. In addition, the sub-microscopic prevalence in our study varied with respect to the study district, being lower (59.8%) in the rural area compared to the urban areas (73%). The high prevalence of sub-microscopic infections observed in the present study, as well as in other studies [19, 30]**,** represents a major challenge to malaria control programs in the Republic of the Congo since the management of malaria patients is based on the microscopic detection of parasites. Our findings suggest that despite the best efforts of the national malaria program, malaria transmission will continue to be high because half of infected individuals remain afebrile and will, therefore, not be identified as having malaria and subsequently treated. Among the non*-P. falciparum* identified by PCR, *P. malariae* and *P. ovale* were detected in 14.9% and 5.3% of participants, respectively. In terms of co-infection, participants co-infected with *P. falciparum/P. malariae* (8.3%) were the most prevalent, followed by participants co-infected with *P. falciparum/P. ovale* (2.6%). It is notable that although non*-P. falciparum* parasites were hardly detected by microscopy, the high rates of sub-microscopic detection of these parasites translate into perennial transmission of these species in endemic areas, thereby constituting a major challenge to achieving malaria eradication. The presence of persons with *P. ovale* and *P. malariae* co-infected with *P. falciparum* in this study highlights the impact of those two latter parasites in asymptomatic and chronic malaria infection. This becomes more critical with triple infection, which was detected at a sub-microscopic prevalence of 1.7% in the present study. *Plasmodium malariae* and *P. ovale* are rarely associated with severe cases of malaria, but their persistence in the body remains a great challenge for malaria eradication. Although *P. malariae* does not cause malarial relapse from persistent liver-stage parasites, recrudescence of blood-stage parasites can occur after long periods without signs or symptoms, even after the infected person has left the endemic region [17, 18]. Even with appropriate treatment, chronic sub-clinical *P. malariae* infection can occur because of its extended pre-patent period when inadequate drug levels in the blood cannot eliminate newly emerging merozoites [41, 42]. *Plasmodium ovale* is responsible for malaria relapses, even after months or even years without the person showing symptoms, due to the presence of hypnozoites [11, 12]. In the present study, the urban setting was revealed as a zone of higher perennial sub-microscopic infection, compared to the rural area where the non-*P. falciparum* species were more pronounced during the dry season. The reason for this association remains unclear. The small numbers of non-*P. falciparum* species detected in our study is a limitation to this study, especially in sub-analyses on demography or other clinical factors.

## Conclusions

The results of this study highlight the need to include non*-P. falciparum* species in malaria control programs, surveillance measures and eradication strategies in the Republic of the Congo. Therefore, a continuous systematic prevention promoting the use of bed nets and intermittent treatment of pregnant women, screening and treatment of* Plasmodium*-infected individuals living in high-transmission settings may strengthen malaria intervention measures.

## Supplementary Information

**Additional file 1: Table S1.** List of primers used for the nested PCR.

## Data Availability

All data are fully available without restriction. Data are available from the FCRM Institutional Data Access. All request for Data should be addressed to the Executive Director of FCRM reachable by the following address Prof. Francine Ntoumi, Villa D6, Cité OMS-Djoué, Brazzaville, République du Congo (Tel. +242-06-9977980, email: francine.ntoumi@uni-tuebingen.de.

## Abbreviations

ACT: Artemisinin-based combination therapy
CeRMI: Centre de recherche sur les maladies infectieuses—Christoph Merieux
HPF: High power field
RR: Relative risk
