# Supplementary material for: Prevalence of non-Plasmodium falciparum species in southern districts of Brazzaville in The Republic of the Congo
Source: Parasit Vectors. 2022 Jun 16;15:209. doi: 10.1186/s13071-022-05312-9 (PMC9200623; doi:10.1186/s13071-022-05312-9)
Supplement: Supplementary file 1 — Additional file 1: Table S1. List of primers used for the nested PCR. [file 13071_2022_5312_MOESM1_ESM.doc]

**Additional file 1**

**TableS1:** list of Primers used for the Nested PCR

| **PCR step** | **plasmodium** | **Oligo Name** | **sequences** |
| --- | --- | --- | --- |
| ***Nested PCR-1*** | ***Plasmodium spp*** | rPLU6 | 5’-TTA AAA TTG TTG CAG TTA AAA CG-3’ |
|  |  | rPLU5 | 5’-CCT GTT GTT GCC TTA AAC TTC-3’ |
| ***Nested PCR-2*** | ***P. falciparum*** | rFAL1 | 5’-TTA AAC TGG TTT GGG AAA ACC AAA TAT ATT-3’ |
|  |  | rFAL2 | 5’-ACA CAA TGA ACT CAA TCA TGA CTA CCC GTC-3’ |
|  | ***P. malarae*** | rMAL1 | 5’-ATA ACA TAG TTG TAC GTT AAG AAT AAC CGC-3’ |
|  |  | rMAL2 | 5’-AAA ATT CCC ATG CAT AAA AAA TTA TAC AAA-3’ |
|  | ***P. ovale*** | rOVA1 | 5’-ATC TCT TTT GCT ATT TTT TAG TAT TGG AGA-3’ |
|  |  | rOVA2 | 5’-GGA AAA GGA CAC ATT AAT TGT ATC CTA GTG-3’ |
|  | ***P. vivax*** | VIV-1 | 5’-CGC TTC TAG CTT AAT CCA CAT AAC TGA TAC-3’ |
|  |  | VIV-2 | 5’-ACT TCC AAG CCG AAG CAA GCA AAG AAA GTC CTT A-3’ |
